# Antibiotic Usage and Healthcare-Associated *Clostridioides difficile* in Patients with and Without COVID-19: A Tertiary Hospital Experience

**DOI:** 10.3390/antibiotics14030303

**Published:** 2025-03-15

**Authors:** Darko Zdravkovic, Ljiljana Markovic-Denic, Vladimir Nikolic, Zoran Todorovic, Marija Brankovic, Aleksandra Radojevic, Dusan Radovanovic, Borislav Toskovic

**Affiliations:** 1 University Clinical Hospital Center Bezanijska Kosa, 11000 Belgrade, Serbia; zdravkovic.darko@kosa.edu.rs (D.Z.); zoran.todorovic@med.bg.ac.rs (Z.T.); brankovic.marija@bkosa.edu.rs (M.B.); radojevic.aleksandra@bkosa.edu.rs (A.R.); radovanovic.dusan@bkosa.edu.rs (D.R.); toskovic.borislav@bkosa.edu.rs (B.T.); 2 Faculty of Medicine, University of Belgrade, 11000 Belgrade, Serbia; 3 Faculty of Medicine, Institute of Epidemiology, University of Belgrade, 11000 Belgrade, Serbia; vladimir.nikolic@med.bg.ac.rs; 4 Faculty of Medicine, Institute of Pharmacology, University of Belgrade, 11000 Belgrade, Serbia

**Keywords:** COVID-19, healthcare-associated infections, *Clostridioides difficile*, antibiotics

## Abstract

**Background/Objectives**: Data about the relationship between COVID-19 and healthcare-associated *Clostridioides difficile* infection (HA-CDI) occurrence are still controversial. This study examines antibiotics associated with CDI in patients with and without COVID-19 infection. **Methods**: A prospective cohort study was conducted at the University Clinical Center Belgrade, Serbia, from January 2019 to December 2021. Patients with the first episode of HA-CDI without and with COVID-19 were included. Results of bacteriology analyses, demographic and clinical data, and data on antibiotic usage and daily defined doses (DDD) were collected by the hospital Infection Control Team. **Results**: Out of 547 HA-CDI cases, 341 (62.3%) had COVID-19 infection. HA-CDI patients with COVID-19 were significantly younger (*p* = 0.017) with fewer comorbidities (<0.001). Two or more antibiotics in therapy were more frequently used by those patients (*p* = 0.03). COVID-19 patients were treated significantly more by third- and fourth-generation cephalosporins, fluoroquinolones (*p* < 0.001) and macrolides (*p* = 0.01). Ceftriaxone had a higher median DDD in COVID-19 patients (6.00, range 1.00–20.00) compared to non-COVID-19 patients (4.00, range 1.00–14.00), (*p* = 0.007). Conversely, meropenem showed a lower median DDD in COVID-19 patients. Multivariate analysis identified the use of fourth-generation cephalosporins and fluoroquinolones as independent risk factors for HA-CDI in COVID-19 patients. **Conclusions**: Patients with HA-CDI and COVID-19 more frequently received two or more antibiotics before the onset of HAI-CDI. The third and fourth generations of cephalosporins, fluoroquinolones and macrolides were administered significantly more often in these patients. More frequent administration of ceftriaxone was observed, but the lower DDD associated with meropenem needed additional analysis.

## 1. Introduction

*Clostridioides difficile* infections (CDIs) are one of the most frequent healthcare-associated infections that contribute to excess morbidity and mortality of hospitalized patients and costs for the healthcare system [1]. Developed countries were the first to recognize the impact of *C. difficile* infections [2,3]. Although the rates may appear falsely low in low- and middle-income countries, they are approaching those in developed countries. Less frequent testing, availability of antibiotics without a prescription, improper use of antibiotics, lack of stewardship and surveillance programs, and lower adherence to hand hygiene protocols contribute to an even greater risk of developing *C. difficile* infection in these populations [4]. The World Health Organization (WHO) declared the COVID-19 pandemic in March 2020 [5]. Hospitals around the world faced many challenges during this pandemic, including the increase in healthcare-associated infections, of which CDIs are one of the most significant, not only in terms of the number of patients but also in the outcome of treatment for patients with COVID-19 and CDI coinfections [6,7]. It was revealed in a recent meta-analysis that 1% of COVID-19 patients develop CDI [8]. Several studies have pointed out that infection prevention and control (IPC) measures were strictly enforced during the COVID-19 pandemic, particularly hand hygiene and the use of gloves and gowns [8]. On the other hand, there has been an increase in the use of antibiotics in COVID patients [9,10], even though COVID-19 is not a bacterial infection and there is no need for antibiotic treatment if there are not bacterial complications [11,12]. However, data about the relationship between COVID-19 and CDI occurrence are still controversial. In some studies, an increased risk of CDI was revealed during the COVID-19 pandemic [6,13,14], while in others, reduced or unchanged CDI rates were observed during the COVID-19 pandemic compared with the pre-pandemic period [8]. CDI development is greatly influenced by prior antibiotic therapy. While it is well known that cephalosporins, fluoroquinolones and clindamycin are the most commonly identified antibiotics associated with CDI, few studies have examined the difference in prior antibiotic use in COVID and non-COVID patients.

This study aims to examine antibiotics associated with healthcare-associated *C. difficile* infection (HA-CDI) in patients with and without COVID-19 infections.

## 2. Results

During 2019, 523 stool samples for *C. difficile* testing were analyzed (5.6 per 1000 patient-days) of which 113 (21.6%) were positive. In 2020, 484 stool samples were analyzed (6.9 per 1000 patient-days) of which 125 (25.8%) were positive, while in 2021, 863 stool samples were analyzed (13.0 per 1000 patient-days) of which 326 (37.8%) were positive. Out of all the positive CDIs, 547 HA-CDI cases were enrolled in the study, of which 341 (62.3%) were SARS-CoV-2-infected patients. The demographic and clinical characteristics of included patients are presented in Table 1. Out of all the enrolled patients, 51.7% were female. The patients with SARS-CoV-2 infection (COVID-19 patients) were statistically younger than those without COVID-19 (69.9 vs. 72.5 years). The statistically higher percentage of patients without COVID-19 disease had comorbidities, with one-quarter of them having three or more comorbidities present (*p* < 0.001). Statistical analysis indicated significant differences when it came to the season in which CDI was diagnosed (*p* < 0.001), with 45% of all patients and 66% of COVID-19 patients presenting in autumn. Non-COVID-19 patients were mostly infected in spring and summer. Among non-COVID-19 patients, the highest proportion were treated in the pulmonology department (36.4%), surgical or intensive care units (24.8%) and cardiology (14.1%). The most common diagnoses for antibiotic prescription in non-COVID patients were pneumonia (30.1%, 62/206), urinary tract infections (18.4%, 38/206), exacerbation of obstructive chronic pulmonary disease (11.7%, 24/206), sepsis (10.7%, 22/206) and postoperative wound infections (4.9%, 10/206), while systemic inflammatory response with no clear anatomical site was recorded in 4.4%.

The use of antibiotics by class is presented in Table 2. Two or more antibiotics in therapy were more frequently used in the COVID-19 patients (*p* = 0.03). COVID-19 patients were treated significantly more by third- and fourth-generation cephalosporins, fluoroquinolones (*p* < 0.001) and macrolides (*p* = 0.01). Also, the duration of treatment with third-generation cephalosporins was significantly longer in COVID-19 patients (*p* = 0.01). Aminoglycosides and other antibiotics were used significantly more in non-COVID-19 patients (*p* < 0.001).

In the multivariate logistic regression analysis, several factors remained significantly associated with HA-CDI in COVID-19 patients (Table 3). Patients with two comorbidities (OR: 0.34, 95% CI: 0.16–0.72, *p* < 0.05) and three or more comorbidities (OR: 0.20, 95% CI: 0.09–0.47, *p* < 0.05) were less likely to be COVID-19 patients with HA-CDI. Seasonality was a strong predictor of HA-CDI in COVID-19 patients, with cases being significantly more frequent in autumn (OR: 9.55, 95% CI: 4.69–19.44, *p* < 0.001), while summer showed a decreased likelihood (OR: 0.35, 95% CI: 0.17–0.73, *p* < 0.05) compared to winter. Regarding antibiotic exposure, the use of fourth-generation cephalosporins (OR: 6.13, 95% CI: 2.47–15.25, *p* < 0.001) and fluoroquinolones (OR: 2.00, 95% CI: 1.21–3.31, *p* < 0.05) was independently associated with HA-CDI in COVID-19 patients.

The analysis of antibiotic use revealed statistically significant differences in the median DDD for ceftriaxone and meropenem between non-COVID-19 and COVID-19 patients (Table 4). Ceftriaxone had a higher median DDD in COVID-19 patients (6.00, range 1.00–20.00) compared to non-COVID-19 patients (4.00, range 1.00–14.00), with a significant *p*-value of 0.007. Conversely, meropenem showed a lower median DDD in COVID-19 patients (3.00, range 1.00–13.00) compared to non-COVID-19 patients (5.50, range 4.00–15.00), with a *p*-value of 0.011.

Ceftriaxone (364.5), Levofloxacin (222.2), and Cefepime (142.3) had the highest overall days of therapy (DOT) per 1000 patient-days (Table 5). During the COVID-19 period, Azithromycin (46.8 → 129.2), Cefepime (28.8 → 214.9), and Levofloxacin (45.4 → 335.0) saw significant increases, reflecting their widespread use. In contrast, Ciprofloxacin (104.2 → 28.0) and Metronidazole (117.2 → 27.4) declined.

## 3. Discussion

Antibiotic use is a well-known modifiable risk factor for healthcare facility-associated *C. difficile* infection. Findings from systematic reviews and meta-analyses have shown that antibiotics from the cephalosporin, fluoroquinolone and clindamycin classes are frequently associated with the development of HA-CDI [16,17]. The World Health Organization (WHO) recently highlighted the extensive overuse of antibiotics during the COVID-19 pandemic, although less than 10% of hospitalized COVID patients had a concomitant bacterial infection that required antibiotic treatment [18]. Our study revealed that a significantly higher percentage of COVID patients received two or more antibiotics in comparison with non-COVID patients. The association between the administration of multiple antibiotics and CDI is supported by clear evidence in the literature, from the first meta-analysis concerning antibiotic use and risk of CDI published in 1998 [19] to today [20,21]. A combination of antibiotics more likely disrupts the balance of normal intestinal flora, allowing *C. difficile* to colonize the large intestine, which could lead to infection [22]. It was found that prior use of more than three antimicrobial agents was an independent risk factor for CDI [23].

According to the European Centre for Disease Prevention and Control (ECDC), cephalosporins were the second (28%) most used subgroup of antibiotics in hospitals at the EU/EEA level in 2023 [24], with variations ranging from 11% (Denmark and Malta) to 61% (Bulgaria). During the period 2013–2022, no significant changes in trends were detected [25]. Although bacterial pneumonia as a complication is much less common with COVID-19 than with influenza [26], antibiotics were often prescribed to patients with COVID-19, at least at the beginning of the pandemic [27]. According to the findings in the recent systematic review and meta-analysis, carbapenems and third- and fourth-generation cephalosporins are antibiotics that are the most strongly associated with HA-CDI, while a modest association was observed for fluoroquinolones [17].

In our study, more than two-thirds of patients with CDI previously received third-generation cephalosporins followed by fluoroquinolones in about 40% of them, fourth-generation cephalosporins (12%) and macrolides (11%), with a significantly higher proportion of COVID patients (76%, 47%, 18% and 14%, respectively). Further, the use of fourth-generation cephalosporins and fluoroquinolones was an independent risk factor for CDI. Cephalosporins are frequently prescribed antibiotics in the medical treatment of patients [28,29]. The majority of our non-COVID patients with CDI were treated in the pulmonology department, with pneumonia being the most common indication for antibiotic use. In COVID-19 patients, pneumonia was a common reason for hospitalization, and third-generation cephalosporins are commonly prescribed for treating it.

Both empirical and directed antimicrobial therapy are used in our hospital according to international guidelines and national protocols. In certain patients, prolonged empirical antibiotic use is necessary due to negative microbiological results (samples are sterile). However, clinical pictures and biomarkers may help in decision making in those cases. Definitive therapy was administered when the lab results were received.

A cohort that included 2356 patients with CDI confirmed the relationship between prior antibiotic use and CDI risk and identified second- and third-generation cephalosporins as the antibiotics that are most important for developing this disease [30]. As in our study, higher use of macrolide during the COVID-19 pandemic was recorded in another hospital in our county [31] as well as in neighboring countries [32]. Analyzing data from 70 countries or territories in the world, Chen et al. [33] confirmed that a constant trend of increasing the global burden of *C*. *difficile* infection (CDI) during recent decades strongly correlates with worldwide antibiotic consumption. In particular, they showed that the consumption of antibiotics (in DDD/1000 inhabitants) correlated with the mortality rate of CDI standardized by age (age-standardized death rate, ASDR) between 2000 and 2015. Interestingly, a similar correlation of ASDR with other factors was lacking: unsafe water, lack of hand-washing facilities, poor hand hygiene, low coverage of health service, low hospital beds and a low number of specialized doctors or nurses. The risk of CDI was highest for people over 67.5 years old who take penicillin, carbapenem, cephalosporins, tetracyclines, macrolides and fluoroquinolones, while the risk of clindamycin was highest for people under 67.5 years old.

Skjøt-Arkil et al. [34] recently assessed the carrier prevalence of *C. difficile* in emergency departments and the association of prior antibiotic consumption in Denmark. Of the 5039 participants, 89 were colonized with *C. difficile* (prevalence of 1.8%). A significant exposure-dependent association between exposure to penicillins and fluoroquinolones and colonization with C. difficile was shown: [DDD/person-year (PY) > 20; OR 4.93 (95% CI 2.22–10.97)], and [DDD/PY > 20; OR 8.81 (95% CI 2.54–30.55)], respectively. At the same time, no such association was found for macrolides.

In a study conducted in two university hospitals during the non-COVID-19 and COVID-19 periods, it was observed that the total antibiotic consumption expressed in DDD did not differ. Conversely, there were significant increases in cephalosporins and carbapenem usage [35]. A significant increase in the use of third-generation cephalosporins by patients with CDI during the COVID-19 period was observed in other studies too [36]. Accordingly, the present results are only partially consistent with data from the literature (Table 4). More frequent application, i.e., greater exposure to ceftriaxone in COVID-19 patients with CDI, is expected, but the opposite result for meropenem is an unexpected finding. According to the national guidelines, carbapenems are usually not the first-line antibiotics for community-acquired pneumonia. However, using meropenem was higher in non-COVID-19 patients, while using imipenem/cilastatin was quite the opposite. This can be explained by the judicious use of the latter antibiotic in our COVID-19 patients, but additional analysis is needed. Although the difference in ceftriaxone and meropenem duration between COVID-19 and non-COVID-19 patients in days is small, a large sample size can make even small differences statistically significant. The question remains whether it is necessary to prescribe antibiotics to such COVID-19 patients [27]. Lewandowski et al. revealed that antibiotics were administered to 80% of COVID-19 patients. Daily antibiotic intake per 100 person-days of hospitalization increased from 57.2 in the pre-pandemic period to 105 during the pandemic [37].

In our study, the number of patients tested for CDI per 1000 patient-days increased 2.3 times from the pre-pandemic year to pandemic years, i.e., from 5.6 per 1000 patient-days in 2019 to 6.9 in 2020 and 13.0 in 2021. At the same time, the percentage of CDI positives increased from 21.6% to 25.8% and 37.8%, respectively. Our results are consistent with the Maldonado-Barrueco et al. study, in which an increase in HO-CDI requests during the pandemic was observed despite a decrease in admissions and patient-days [38]. In contrast, in a large cohort study conducted in 772 US hospitals, although *C. difficile* testing rates were higher in the pandemic year than in the pre-pandemic year, the percentage of HA-CDI positives did not change [39].

We found nearly an equal percentage of male and female patients with CDI/COVID-19 co-infection, contrary to the findings of a university hospital in the northern part of our country [31], as in other countries [37] that reported a higher percentage of male patients. Further, our patients with CDI/COVID-19 co-infection were younger and had fewer comorbidities. The younger age of COVID-19 patients can be explained by more hospitalization of young people due to severe pneumonia caused by the Delta variant of SARS-CoV-2 in the first years of the pandemic [40] and the “UK-variant”, i.e., 20I/501Y.V1 [41]. Multivariate analysis in our study revealed a significantly lower presence of comorbidities in COVID-19 patients. Younger people tend to have fewer comorbidities. Besides other factors, seasonality was also an independent predictor of HA-CDI in COVID-19 patients, with significantly more cases in autumn. The increase in HA-CDI coincides with the rise in COVID-19 in the population and in-hospital treatment, which was observed in other studies [42]. During COVID-19 management in our hospital, an increased use of antibiotics was observed. The dedication of the hospital to COVID-19 care during the 11 months of the study period, while coinciding with the pattern of illness in the community, may have served as an additional limitation of our study.

Another limitation of this study is that antibiotic administration before hospital admission was not monitored. These antibiotics may be received in different inpatient admissions or ambulatory settings, and data about them are not always available. Our analysis focused only on antibiotic use within our hospital, available in the patient’s medical records and the hospital information system.

## 4. Materials and Methods

A prospective cohort study was conducted at the University Clinical Center Bezanijska Kosa, Belgrade, Serbia, from January 2019 to December 2021. The study began as hospital comprehensive surveillance before the COVID pandemic because it was essential to recognize antibiotic usage among *C. difficile* risk factors. It continued throughout the COVID pandemic period. Our hospital is one of five teaching hospitals in the capital where it provides healthcare to the largest municipality, which has approximately 210 thousand inhabitants. There are 360 beds in the hospital, 33 of which are in intensive care units. The hospital has surgical and medical wards, but no neurology, gynecology and pediatric wards. The hospital treated about 18 thousand patients with over 90 thousand patient-days during the pre-COVID year and an average of 10 thousand patients with 68 thousand patient-days during the COVID pandemic period.

In Serbia, the first COVID-19 case was confirmed on 6 March 2020, and then the four waves of the COVID-19 epidemic in 2020–2021 were observed [43].

This hospital served as a COVID-dedicated hospital during the period of 12 months (from July 2020 to December 2021 except for the periods February-March, and May-September 2021). During the two aforementioned periods, the stagnation of new cases of COVID-19 in Serbia was recorded. Only non-COVID patients were hospitalized for the remainder of the observed period of 24 months. During the period when the hospital was COVID-dedicated, only COVID-19 patients were treated in all departments. Operations were rare and only in COVID-19 patients who required urgent surgical intervention. In addition to the existing beds in intensive care units (ICUs), another ward was provided with an oxygen supply, new respirators were purchased and the ward was transformed into an ICU. Throughout the hospital, the staff strictly wore personal protective equipment (PPE), and dedicated areas for PPE donning and doffing were prepared and equipped.

Patients with the first episode of CDI were included. Clinical criteria and laboratory confirmation were used to diagnose CDI, following the ECDC recommendations, which were translated into Serbian (MoH). Only if the patient had three or more unformed stools in 24 h, a stool sample was sent for laboratory testing. Initial testing with glutamate dehydrogenase (GDH) was performed. Then, positive GDH results were confirmed by an enzyme-linked immunosorbent assay (ELISA) test for detecting toxins A and/or B in stools [44,45,46]. The CITEST^®^, CITEST DIAGNOSTICS Inc., Vancouver, BC, Canada, and VIDAS^®^ C. difficile Toxin A&B (CDAB), bioMérieux SA (Durham, NC, USA) were used. The COVID-19 infection diagnosis was based on clinical criteria and a positive nasopharyngeal swab polymerase chain reaction (PCR) test [47,48].

### 4.1. Data Collection

The Hospital Microbiology laboratory reports daily on the hospital information system regarding the results of all bacteriology analyses, including testing for *C. difficile*. Furthermore, in the event of CDI-positive results, a quick phone call was made to the Department for Infection Prevention. The hospital epidemiologist analyzed laboratory and clinical data to determine if healthcare-associated *C. difficile* infection (HA-CDI) was present. The ECDC definitions for HAI translated into the Serbian language were used [49]. Data about age, gender, comorbidities, results of PCR testing, antibiotic usage in the hospital before CDI diagnostics, and outcome were collected. Data on antibiotic use included generic names, class of antibiotics, and defined daily dose—DDD [50]. In our hospital, antibiotics are administered according to national guidelines for the rational use of antibiotics [51]. Comorbidities recorded in this study included hypertension, coronary artery disease, chronic obstructive pulmonary disease, advanced-stage renal insufficiency, malignancy, inflammatory bowel disease, history of stroke, chronic liver disease, endocrine disorders and diabetes mellitus.

### 4.2. Statistical Analysis

The local infection prevention and control (IPC) team entered all data into the previously prepared database. Continuous variables were presented as mean ± standard deviation (SD) or median (minimum-maximum) as appropriate, depending on data distribution. Categorical variables were expressed as frequencies and percentages. The normality of continuous variables was assessed using the Shapiro–Wilk test. Comparisons of continuous variables between groups (COVID-19 vs. non-COVID-19 periods) were performed using the independent samples *t*-test for normally distributed data or the Mann–Whitney U test for data without normal distribution. Categorical variables were compared using the Chi-square test. A *p*-value of <0.05 was considered statistically significant for all tests. DDDs were calculated for each antibiotic by summing the total amount of the drug administered (in grams) divided by the WHO-assigned standard DDD value for that specific antibiotic. Days of therapy (DOT) per 1000 patient-days was calculated by counting each antibiotic that each patient was prescribed and expressed in 1000 patient-days. Data analysis was performed using SPSS version 17.0 software (SPSS Inc., Chicago, IL, USA).

## 5. Conclusions

Patients with HA-CDI and COVID-19 were younger with less comorbidity than non-COVID-19 patients. They more frequently received two or more antibiotics before the onset of HAI-CDI. The third and fourth generations of cephalosporins, fluoroquinolones and macrolides were administered significantly more often in these patients. More frequent administration of ceftriaxone, i.e., higher median DDD in COVID-19 patients, was observed. The lower DDD found for meropenem needed additional analysis.

## Figures and Tables

**Table 1:** Demographic and clinical characteristics of patients with *C. difficile* infection [15].

	Total n (%)	Non-COVID-19 n (%)	COVID-19 n (%)	*p* Value
Age	70.9 ± 12.3	72.5 ± 11.6	69.9 ± 12.6	
Age under 65 years	133 (24.3)	38 (18.4)	95 (27.9)	**0.017**
Age 65 years and above	414 (75.7)	168 (81.6)	246 (72.1)	
Sex				
Female	283 (51.7)	110 (53.4)	173 (50.7)	0.546
Male	264 (48.3)	96 (46.6)	168 (49.3)	
BMI	27.5 ± 5.5	27.6 ± 6.9	27.5 ± 4.7	0.900
Comorbidity				
Without	97 (17.1)	16 (7.8)	81 (23.8)	**<0.001**
One comorbidity present	177 (32.4)	57 (27.7)	120 (35.2)	
Two comorbidities present	184 (33.6)	79 (38.3)	105 (30.8)	
Three or more comorbidities present	89 (16.3)	54 (26.2)	35 (10.3)	
Season during the medical examination				
Spring	117 (21.4)	73 (35.4)	44 (12.9)	**<0.001**
Summer	92 (16.8)	68 (33)	24 (7)	
Autumn	246 (45)	21 (10.2)	225 (66)	
Winter	92 (16.8)	44 (21.4)	48 (14.1)	
Outcome				
Discharge	427 (77.7)	155 (72.5)	270 (79.2)	0.387
Death	121 (22.1)	81 (24.8)	70 (20.5)	
Admission to another hospital	1 (0.2)	1 (0.3)	0 (0.0)	
Duration of hospitalization	20.5 ± 11.8	21.2 ± 12.8	20.1 ± 11.2	0.272

**Table 2:** Antibiotic usage and duration (days) in patients with *C. difficile* infection.

Antibiotics	Total		Non-COVID-19		COVID-19		*p* Value
	n (%)	Duration Mean ± SD	n (%)	Duration Mean ± SD	n (%)	Duration Mean ± SD	
Cephalosporins 2nd gen	6 (1.1)	4.2 ± 2.5	4 (1.9)	4.7 ± 2.5	2 (0.6)	2.0 ± 0.0	0.205 *, 0.398 **
Cephalosporins 3rd gen	378 (69.1)	7.1 ± 4.8	119 (57.8)	6.1 ± 3.7	259 (76.0)	7.5 ± 5.1	<0.001 *, 0.010 **
Cephalosporins 4th gen	68 (12.4)	5.6 ± 3.4	7 (3.4)	7.7 ± 2.6	61 (17.9)	5.7 ± 3.5	<0.001 *, 0.472 **
Aminoglycosides	33 (6.0)	6.1 ± 4.4	23 (11.2)	6.2 ± 4.2	10 (2.9)	5.9 ± 5.2	<0.001 *, 0.873 **
Fluoroquinolones	208 (38)	7.0 ± 4.4	49 (23.8)	6.5 ± 3.6	159 (46.6)	7.2 ± 4.6	<0.001 *, 0.352 **
Sulfonamides and trimethoprim	15 (2.7)	6.1 ± 4.4	9 (4.4)	6.1 ± 5.1	6 (1.8)	6.1 ± 4.0	0.070 *, 0.994 **
Macrolides, lincosamines and streptogramins	60 (11.0)	5.7 ± 4.0	11 (5.3)	6.2 ± 5.4	49 (14.4)	5.5 ± 3.7	0.010 *, 0.642 **
Carbapenems	92 (16.8)	7.5 ± 5.5	31 (15.0)	8.4 ± 5.2	61 (17.9)	7.0 ± 5.7	0.390 *, 0.277
Tetracyclines and chloramphenicol	6 (1.1)	5.0 ± 1.9	1 (0.5)	NA	5 (1.5)	5.0 ± 1.9	0.286 *, NA **
Glycopeptides	33 (6.0)	6.9 ± 4.5	17 (7.3)	7.9 ± 4.7	18 (5.3)	6.1 ± 4.3	0.340 *, 0.288 **
Polymyxins	22 (4.0)	7.0 ± 5.8	8 (3.9)	4.4 ± 1.7	14 (4.1)	8.0 ± 6.5	0.898 *, 0.242 **
Other antibiotics	43 (7.9)	8.4 ± 5.4	29 (14.1)	9.21 ± 5.90	14 (4.1)	7.00 ± 4.32	<0.001 *, 0.244 **
The number of applied groups of antibiotics							
One	151 (30.0)		64 (38.1)		87 (25.9)		0.03 *
Two	173 (34.3)		55 (32.7)		118 (35.1)		
Three	105 (20.8)		30 (17.9)		75 (22.3)		
Four and more	75 (14.9)		19 (11.3)		56 (16.7)		

* Chi-square test comparing the frequency of antibiotic use between non-COVID-19 and COVID-19 patients; ** independent samples *t*-test comparing the duration of antibiotic use between non-COVID-19 and COVID-19 patients.

**Table 3:** Logistic regression analysis of factors associated with HA-CDI in COVID-19 patients.

	Univariate Logistic Regression OR (95% CI)	Multivariate Logistic Regression OR (95% CI)
Age		
Age under 65 years		
Age 65 years and above	0.59 (0.38–0.89)	
Comorbidities		
Without	ref.	ref.
1 comorbidity present	0.42 (0.22–0.77)	0.54 (0.25–1.18)
2 comorbidities present	0.26 (0.14–0.48)	0.34 (0.16–0.72)
3 or more comorbidities present	0.13 (0.06–0.25)	0.20 (0.09–0.47)
Season during the medical examination		
Winter	ref.	ref.
Spring	0.55 (0.32–0.96)	0.64 (0.32–1.26)
Summer	0.32 (0.17–0.60)	0.35 (0.17–0.73)
Autumn	9.82 (5.36–18.01)	9.55 (4.69–19.44)
Cephalosporins 3rd gen	2.31 (1.59–3.35)	
Cephalosporins 4th gen	6.20 (2.78–13.82)	6.13 (2.47–15.25)
Aminoglycosides	0.24 (0.11–0.52)	0.23 (0.09–0.62)
Fluoroquinolones	2.80 (1.91–4.11)	2.00 (1.21–3.31)
Macrolides, lincosamines and streptogramins	2.99 (1.51–5.88)	
The number of applied groups of antibiotics		
One	ref.	
Two	1.58 (1.00–2.49)	
Three	1.84 (1.08–3.13)	
Four and more	2.17 (1.17–4.00)	

Ref.—Reference; OR—Odds ratio; CI—Confidence interval.

**Table 4:** Median number of DDD of the most frequently used antibiotics.

Antibiotic	Total	Non-COVID-19	COVID-19	*p*-Value
	DDD Median (Min–Max)			
Amikacin	4.50 (1.00–6.00)	5.00 (1.00–6.00)	4.00 (2.00–6.00)	1.000
Amoxicillin-clavulanate	9.75 (2.00–14.00)	14.00 (14.00–14.00)	5.50 (2.00–13.00)	0.137
Azithromycin	5.75 (2.00–12.00)	6.50 (5.00–11.00)	5.00 (2.00–12.00)	0.068
Cefepime	5.00 (1.00–13.00)	5.00 (1.00–11.00)	5.00 (1.00–13.00)	1.000
Cefixime	4.50 (2.00–10.00)	5.00 (5.00–5.00)	4.00 (2.00–10.00)	0.573
Ceftazidime	3.50 (1.00–7.00)	3.00 (1.00–5.00)	4.00 (2.00–7.00)	0.437
Ceftriaxone	5.00 (1.00–20.00)	4.00 (1.00–14.00)	6.00 (1.00–20.00)	**0.007**
Cefuroxime	9.50 (2.00–12.00)	7.00 (2.00–8.00)	12.00 (12.00–12.00)	0.276
Ciprofloxacin	5.25 (1.00–10.00)	5.00 (1.00–10.00)	5.50 (1.00–10.00)	0.724
Clindamycin	2.00 (1.00–3.00)	1.00 (1.00–1.00)	3.00 (3.00–3.00)	1.000
Colistimethate-sodium	3.50 (3.00–4.00)	4.00 (4.00–4.00)	3.00 (3.00–3.00)	0.248
Imipenem-cilastatin	5.00 (3.00–8.00)	4.00 (3.00–4.00)	6.00 (4.00–8.00)	0.164
Levofloxacin	4.75 (1.00–20.00)	4.00 (3.00–10.00)	5.50 (1.00–20.00)	0.422
Meropenem	4.25 (1.00–15.00)	5.50 (4.00–15.00)	3.00 (1.00–13.00)	**0.011**
Metronidazole	7.75 (1.00–13.00)	5.50 (1.00–13.00)	10.00 (10.00–10.00)	0.525
Trimethoprim-sulfamethoxazole	7.00 (3.00–10.00)	4.00 (3.00–7.00)	10.00 (10.00–10.00)	0.500
Vancomycin	4.50(4.00–5.00)	4.00 (4.00–4.00)	5.00 (5.00–5.00)	0.479

**Table 5:** Comparison of days of therapy per 1000 patient-days during COVID-19 and non-COVID-19 periods.

Antibiotic	Total	Non-COVID-19	COVID-19
	Days of Therapy Per 1000 Patient-Days		
Amikacin	38.7	45.7	34.3
Amoxicillin-clavulanate	28.9	5.5	43.8
Azithromycin	87.1	46.8	129.2
Cefepime	142.3	28.8	214.9
Cefixime	41.7	2.3	66.9
Ceftazidime	59.6	51.4	64.8
Ceftriaxone	364.5	226.3	452.7
Cefuroxime	7.1	11.9	4.1
Ciprofloxacin	57.7	104.2	28.0
Clindamycin	9.3	6.4	11.2
Colistimethate-sodium	30.4	8.0	44.8
Imipenem-cilastatin	32.1	32.9	31.6
Levofloxacin	222.2	45.4	335.0
Meropenem	108.3	108.1	125.0
Metronidazole	62.4	117.2	27.4
Trimethoprim-sulfamethoxazole	23.0	35.9	14.8
Vancomycin	65.0	82.9	53.5

## Data Availability

The data presented in this study are available on request from the corresponding author due to privacy.

## Abbreviations

The following abbreviations are used in this manuscript:

BMI	Body mass index
CDI	*Clostridioides difficile* infections
COVID-19	Coronavirus disease 2019
DDD	Defined daily dose
ECDC	European Centre for Disease Prevention and Control
ELISA	Enzyme-linked immunosorbent assay
GDH	Glutamate dehydrogenase
HA-CDI	Healthcare-associated *C. difficile* infection
IPC	Infection prevention and control
PCR	Polymerase chain reaction
PY	Person-year
SD	Standard deviation
WHO	World Health Organization
